# Delirium Mistaken for Bipolar Disorder in a Paediatric Oncology Patient: A Case Report

**DOI:** 10.62641/aep.v53i6.2006

**Published:** 2025-12-17

**Authors:** Zhe Shen, Rongwang Yang, Weijia Gao

**Affiliations:** ^1^Department of Child Psychology, The Children’s Hospital, Zhejiang University School of Medicine, National Clinical Research Center for Child Health, National Children’s Regional Medical Center, 310052 Hangzhou, Zhejiang, China

**Keywords:** delirium, paediatric oncology, case report, paediatric bipolar disorder

## Abstract

Delirium is a clinically significant complication in paediatric oncology that often leads to diagnostic delays. This case describes a 12-year-old boy with high-risk B-cell acute lymphoblastic leukaemia who developed acute neuropsychiatric symptoms, including agitation, hallucinations, seizures, and fluctuating mood states. Initially misdiagnosed with paediatric bipolar disorder, the patient was treated with antipsychotics and sedatives with limited effects. Notably, the hyperactive symptoms transitioned to a hypoactive state following the discontinuation of meropenem. This case illustrates the diagnostic challenges in distinguishing delirium from psychiatric disorders in paediatric settings, especially in the context of complex medical treatments. The case highlights the need for greater clinical awareness, routine delirium screening using validated tools, and careful evaluation of the neurotoxic potential of multiple medications in paediatric oncology patients.

## Introduction

Delirium is an acute neuropsychiatric syndrome characterized by fluctuating disturbances in attention, awareness, and cognition. Although it is relatively common among critically ill paediatric patients, it remains significantly underdiagnosed—particularly in specialized paediatric hospitals in developing countries such as China. A cross-sectional survey in paediatric hospitals in China revealed that more than 80% of health care professionals had never performed delirium screening in children, indicating a critical gap in clinical awareness and routine practice [1].

Despite the growing recognition of the clinical impact of delirium, only 26% of ICU staff routinely screen for it, and only 16% use validated tools such as the Confusion Assessment Method for the ICU (CAM-ICU) [2]. This discrepancy between perceived importance and actual practice can lead to frequent misdiagnosis or neglect.

Hypoactive delirium, which presents with withdrawn or subdued behaviour, is especially prone to being mistaken for fatigue or depression and thus is often overlooked [3]. Conversely, in adolescents, hyperactive delirium—marked by agitation and emotional dysregulation—may mimic psychiatric conditions such as bipolar disorder or impulse control disorders, increasing the risk of misdiagnosis.

This case highlights the important topic of the underrecognition of paediatric delirium in clinical settings.

## Clinical Case

**Fig. 1.**
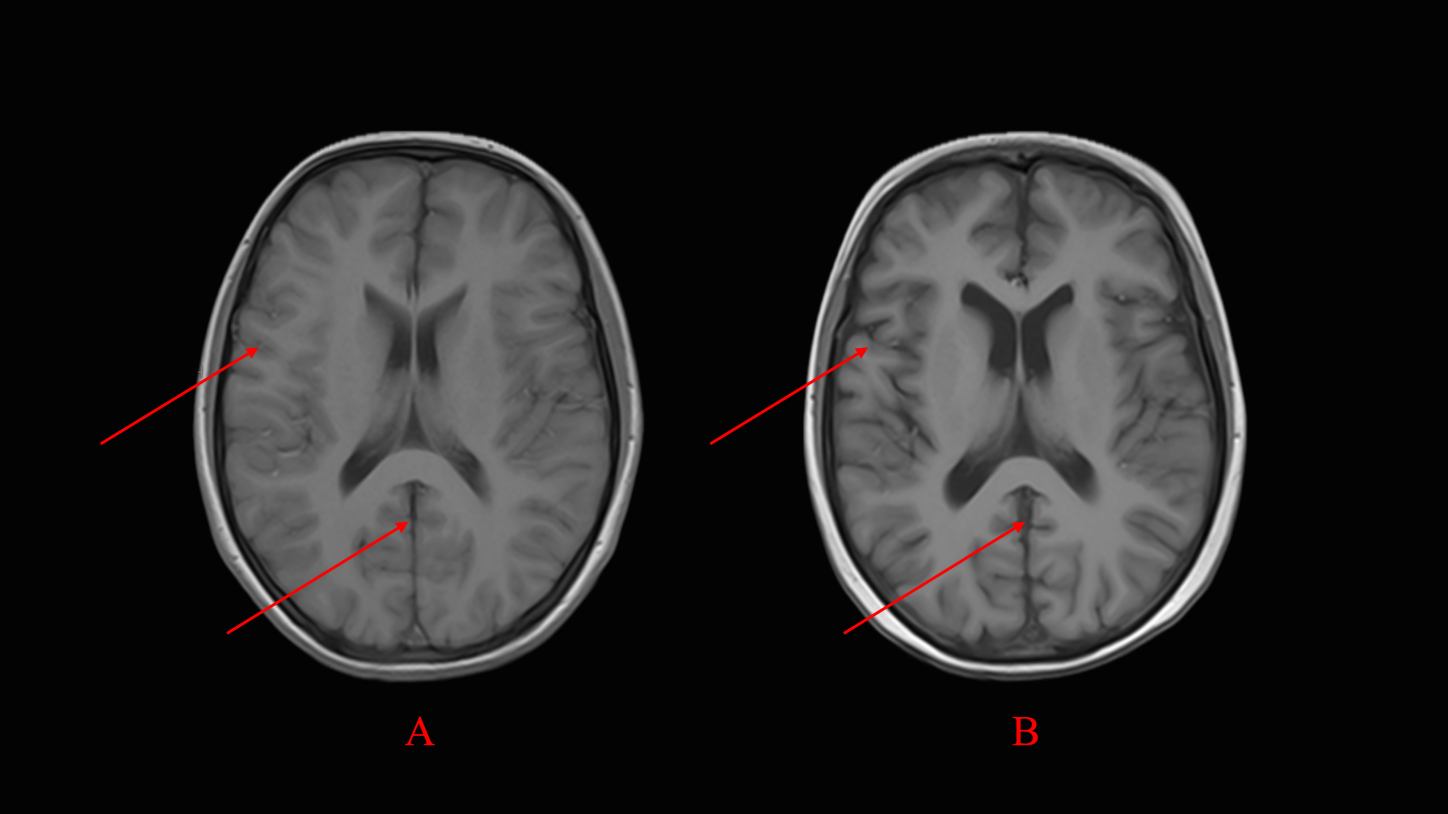
**Progressive cerebral atrophy on follow-up MR image**. (A) Baseline brain MR image showing mild sulcal widening without other significant abnormalities. (B) repeated MR image showing widened sulci and signs of cerebral atrophy (indicated by arrows). MR, Magnetic Resonance.

On August 5, 2023, a 12-year-old boy with high-risk B-cell acute lymphoblastic leukaemia (ALL) was admitted for induction chemotherapy, including vincristine, daunorubicin, l-asparaginase, dexamethasone, and oral dasatinib. Baseline brain Magnetic Resonance Imaging (MRI) revealed mild sulcal widening without other abnormalities (Fig. 1A), and electroencephalography (EEG) showed normal background activity with no epileptiform discharges. The patient had no documented personal or family history of neurological or psychiatric disorders. The manufacturer, lot number, and location for all drugs and instruments used in this study are provided in the **Supplementary Material 1**.

Following the initiation of chemotherapy, the patient developed a low-grade fever (maximum 37.6 °C) and elevated C-reactive protein (CRP, 40.32 mg/L) on Day 5 after admission. Empirical intravenous antibiotic therapy was initiated with meropenem (800 mg every 12 hours), with vancomycin (806 mg every 12 hours) added three days later. Laboratory parameters, including arterial blood gases, electrolytes, infection markers, cytokine panels, and metabolic profiles, were largely within normal limits. There was no evidence of renal impairment or electrolyte disturbance.

On the night of Day 5 after admission, the patient developed acute behavioural disturbances, including insomnia, agitation, incoherent speech, and episodic screaming. He was administered 10 mg of intravenous diazepam for sedation. Three hours later, he experienced two generalized tonic seizures, each lasting approximately one minute and featuring gaze deviation, frothing, and limb rigidity. Postictal EEG revealed generalized background slowing (<8 Hz), and repeat MRI showed accelerated cerebral atrophy compared with baseline (Fig. 1B). Despite evidence of cerebral dysfunction, the seizures were considered a hysterical phenomenon arising from the patient’s agitated state. In view of these acute complications, chemotherapy was then discontinued.

During the subsequent five days, the patient presented with persistent neuropsychiatric symptoms, including nocturnal agitation, visual hallucinations (e.g., reports of strangers in the room), restlessness, and shouting. He also displayed marked hyperexcitability, irritability, and pressured speech, frequently engaging nurses and others in incessant, difficult-to-interrupt conversations. He was managed with intravenous diazepam, midazolam, oral aripiprazole (5 mg twice daily) and valproate (1000 mg at night, 500 mg in the morning), as his clinical presentation was considered consistent with a probable manic episode.

On Day 12 after admission, meropenem was discontinued. Within 24–48 hours, the hallucinations and agitation began to subside. The patient then experienced mutism, psychomotor slowing, and continued insomnia accompanied by low mood, frequent crying, and muttering self-reproaches about being a burden to his family. The patient was suspected to have bipolar disorder on the basis of the observed transition from the manic phase to the depressive phase. However, the ongoing administration of valproate, aripiprazole, and benzodiazepines failed to improve his psychiatric symptoms.

A psychiatric consultation was subsequently arranged. Two experienced psychiatrists established the diagnosis of delirium per the DSM-5 criteria [4], which require an acute disturbance in attention and awareness that fluctuates in severity. The patient presented with an acute (within hours) onset of impaired attention and a reduced level of consciousness, fluctuating markedly over 24 hours, accompanied by acute deterioration in orientation and memory. These changes could not be accounted for by preexisting dementia and were temporally linked to infection and antimicrobial therapy, fulfilling the DSM-5 criteria for delirium. The diagnosis was further objectively confirmed by a retrospective Cornell Assessment of Paediatric Delirium (CAPD) score of 26/32. The combination of ongoing valproate and benzodiazepine use, coupled with the withdrawal of meropenem, is considered to have collectively contributed to the patient’s shift from hyperactive to hypoactive delirium. Consequently, the medication regimen was adjusted: valproate and benzodiazepines were discontinued, and aripiprazole was tapered to 1.25 mg daily.

**Fig. 2.**
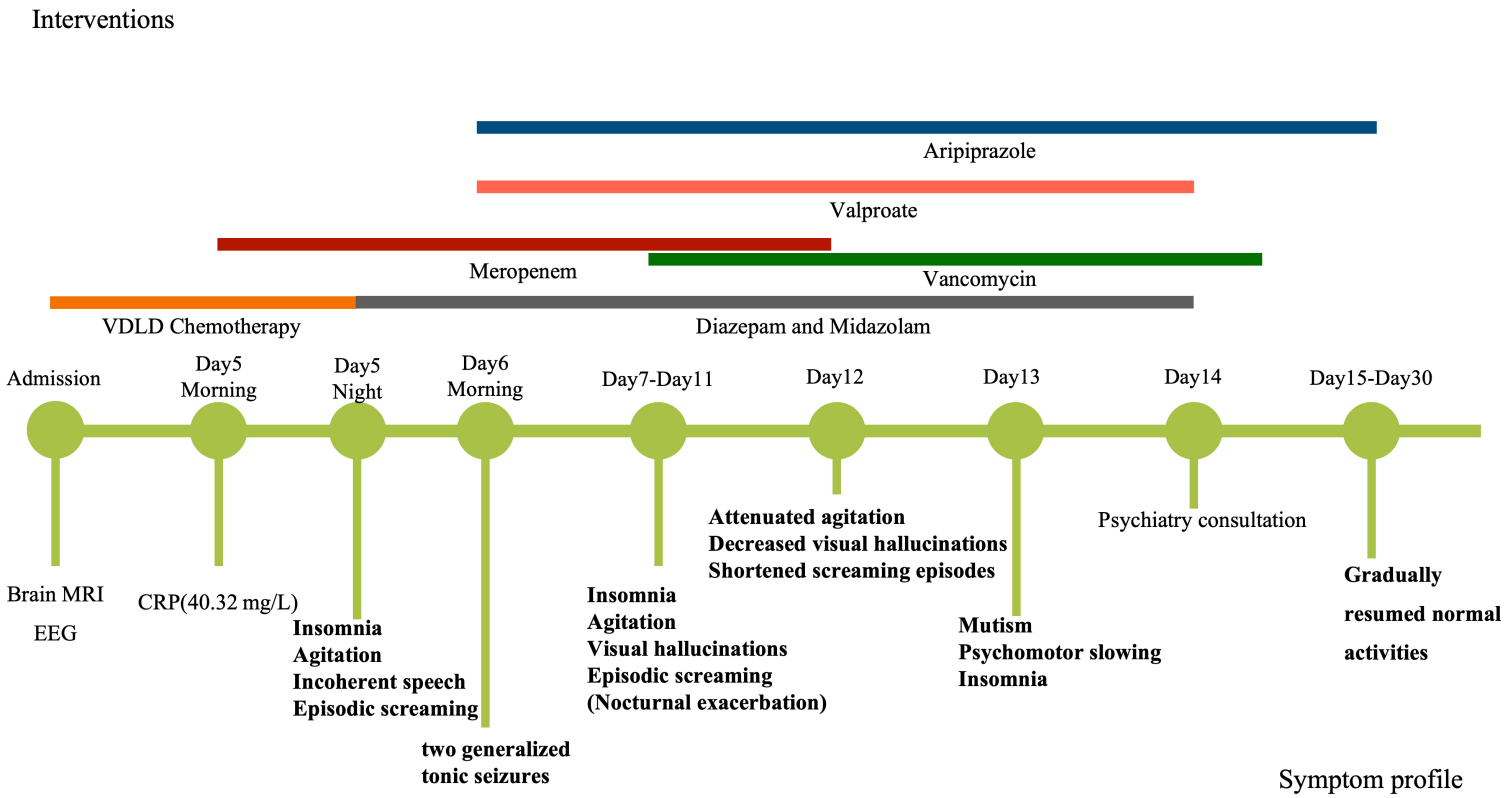
**Clinical timeline**. VDLD, chemotherapy including vincristine, daunorubicin, l-asparaginase and dexamethasone); MRI, Magnetic Resonance Imaging; EEG, electroencephalography; CRP, C-reactive protein.

Over the next two weeks, the patient gradually resumed normal activities, although mild memory impairment and delayed responses persisted. At 30 days post-admission, aripiprazole was discontinued. The patient demonstrated restored emotional stability and resolution of hallucinations. During the six-month follow-up, he successfully completed five chemotherapy cycles without recurrence of neuropsychiatric symptoms. The patient’s clinical course is illustrated in a timeline (Fig. 2).

## Discussion

We describe a case of suspected antibiotic-associated delirium and complex drug interactions in a paediatric high-risk B-cell ALL patient with postchemotherapy infection.

Despite its clinical significance, paediatric delirium is often misunderstood, leading to hyperactive symptoms being misdiagnosed as bipolar or impulse control disorders [5]. In this case, the patient’s acute behavioural disturbances, including agitation, hallucinations, and incoherent speech, were a hallmark of delirium but mimicked a manic episode, leading to initial misdiagnosis. The overlap between delirium and bipolar disorder can complicate the diagnostic process. Both conditions may involve agitation, sleep disruption, irritability, and psychotic symptoms. However, key distinguishing features of delirium include an acute onset, a fluctuating course, impaired attention, and disturbances in attention and awareness—features that are typically absent in primary mood disorders [6]. A retrospective CAPD score of 26 also strongly supported the diagnosis of delirium. In contrast, bipolar disorder is usually characterized by sustained mood disturbance, preserved orientation and attention (at least in the early stages), and a chronic relapsing course with prior episodes. This case is distinctive not only because the initial hyperactive delirium mimicked mania but also because of its uncommon progression to a hypoactive phase, a dynamic course that highlights a clear drug-related causation.

Multiple factors may have contributed to the delirium, and chemotherapeutic agents such as vincristine and L-asparaginase have been associated with neurotoxic effects [7]. The exact mechanisms remain unclear but may involve disruption of the blood–brain barrier and elevated cytokine levels affecting central nervous system (CNS) function, which could have contributed to CNS vulnerability. However, chemotherapy was discontinued because of the patient’s uncooperative behaviour during hyperactive delirium, and the symptoms persisted until the discontinuation of meropenem. Meropenem is generally well tolerated and has a lower incidence of neurotoxicity than other antibiotics do [8], and cases of meropenem-induced delirium have rarely been reported, particularly in the paediatric oncology population. Previous reports identified three meropenem-associated delirium cases (Table 1) [9, 10, 11]. Proposed mechanisms for meropenem-induced neurotoxicity include the inhibition of γ-aminobutyric acid (GABA) receptor-mediated inhibitory transmission [12]. To more objectively evaluate the causal relationship between meropenem and delirium, we used the Naranjo Adverse Drug Reaction Probability Scale [13]. The assessment yielded a score of 8, indicating that meropenem was a ‘probable’ cause of the patient’s delirium. These results support our clinical conclusion that meropenem played a significant role in the neuropsychiatric symptoms observed in this case. Previous reports involved elderly patients with renal impairment [9, 10], whereas our patient was a child with normal renal function and no psychiatric history. In paediatric oncology patients, the combined insult of chemotherapy-induced blood–brain barrier vulnerability and infection-related inflammatory states render them more vulnerable to the pronounced neurotoxic effects of antibiotics. Prior cases of meropenem-induced delirium describe a hyperactive phenotype [9, 10, 11], which is consistent with the early stage of our case. However, our report is the first to document a subsequent transition to hypoactive delirium following meropenem discontinuation.

**Table 1.** **Demographic and clinical data of reviewed patients**.

ID	Sex*	Age	Delirium type	Duration**	Meropenem***	Other medications
Yang *et al*. [9]	F	76	manic; confused; shouted	30 min	1000 mg q8h	Dexmedetomidine; Ceftriaxone
Munoz-Gomez *et al*. [10]	M	100	confused; agitated	24 hours	500 mg q12h	Piperacillin-tazobactam
Tabulov *et al*. [11]	F	15	confused; agitated; hallucinations	2 days	500 mg qd	Olanzapine; Morphine, Lorazepam; Corticosteroids; Piperacillin-tazobactam
Present case	M	12	agitated; manic; hallucinations; mutism	Hypoactive: 8 days	800 mg q12h	Valproate; Benzodiazepines; aripiprazole
				Hyperactive: 2 weeks		

*Sex: Male (M)/Female (F); **Duration: The duration of delirium symptoms, which resolved completely in all patients after meropenem was discontinued. ***Meropenem: The intravenous dosage and dosing frequency of meropenem.

Notably, the administration of valproate and benzodiazepines during meropenem therapy failed to effectively control the patient’s hyperactive delirium. Meropenem likely diminished the therapeutic impact of valproate by reducing its serum concentration [14]. The transition to hypoactive delirium likely involved multifactorial mechanisms. First, compensatory GABA receptor upregulation may have occurred after meropenem withdrawal, at which point the previously masked GABAergic effects of benzodiazepines and valproate became dominant, leading to excessive inhibition [15]. The sedative effects of diazepam and its active metabolites, which have half-lives of 20–100 hours [16], may become fully apparent after the discontinuation of meropenem. Additionally, continued valproate administration may have contributed to excessive sedation [17]. Finally, competitive drug metabolism and neurotransmitter disruption collectively cause deficits in awareness, thinking, and behavioural control.

This case also highlights the importance of the systematic assessment of delirium in clinical paediatric practices. Structured tools such as the CAPD and Prescreening Confusion Assessment Method for the Intensive Care Unit (pCAM-ICU) can provide standardized approaches for identifying fluctuating attention, altered awareness, and cognitive change—features that distinguish delirium from primary psychiatric disorders. When this patient’s clinical course was retrospectively reviewed using the CAPD criteria, it was confirmed that delirium could have been detected earlier. Using these tools, protocolizing screening triggers (e.g., for new-onset agitation or after specific treatments) can help target high-risk populations efficiently.

From a point of education, this case emphasizes that paediatric delirium may mimic mania-like symptoms, leading to potentially harmful misdiagnoses and unnecessary psychotropic treatment. Recognizing key differentiating features of acute onset, fluctuation over time, and the presence of disorientation can guide physicians towards accurate identification. Early involvement of psychiatry and neurology teams can further support accurate diagnosis and timely intervention.

This study has several limitations. First, as a retrospective case review, standardized delirium assessment tools such as the pCAM-ICU or CAPD were not used at the time of evaluation, which may have affected the accuracy of delirium identification and characterization. Second, multiple confounding factors were present in the clinical course, including chemotherapy, infection, and polypharmacy, making it difficult to isolate the specific contribution of meropenem to the observed neuropsychiatric symptoms. The association between antibiotic use and delirium in this case is largely based on clinical judgement rather than definitive evidence. Therefore, further case studies and prospective studies are needed to clarify the causal role of antibiotics in delirium among paediatric oncology patients.

## Conclusion

This case illustrates the diagnostic challenges in differentiating delirium from mood disorders in paediatric oncology practice. First, physicians should maintain a high index of suspicion for delirium whenever sudden behavioural or emotional changes occur in medically ill children, especially during infection or exposure to neuroactive medications. Second, the systematic use of screening instruments, such as the CAPD, is essential for early recognition and accurate differentiation from psychiatric conditions. Third, careful assessment of neurotoxic agents such as meropenem is essential. Attention to polypharmacy is important, as psychotropic medications may exacerbate or prolong delirium. Ultimately, a multidisciplinary approach is vital for improving outcomes in this high-risk population.

## Availability of Data and Materials

The datasets during the current study are available from the corresponding author on reasonable request.

## Supplementary Material

Supplementary material associated with this article can be found, in the online version, at https://doi.org/10.62641/aep.v53i6.2006.

## References

[1] Huang X, Lei L, Zhang S, Yang J, Yang L, Xu M (2021). Implementation of the “awakening and breathing trials, choice of drugs, delirium management, and early exercise/mobility” bundle in the pediatric intensive care unit of tertiary hospitals in southwestern China: a cross-sectional survey. *The Journal of International Medical Research*.

[2] Xing J, Sun Y, Jie Y, Yuan Z, Liu W (2017). Perceptions, attitudes, and current practices regards delirium in China: A survey of 917 critical care nurses and physicians in China. *Medicine*.

[3] Dechnik A, Traube C (2020). Delirium in hospitalised children. *The Lancet. Child & Adolescent Health*.

[4] American Psychiatric Association, DSM-5 Task Force (2013). *Diagnostic and statistical manual of mental disorders: DSM-5™. 5th edn*.

[5] Lee BS, Huang SS, Hsu WY, Chiu NY (2012). Clinical features of delirious mania: a series of five cases and a brief literature review. *BMC Psychiatry*.

[6] Karmacharya R, England ML, Ongür D (2008). Delirious mania: clinical features and treatment response. *Journal of Affective Disorders*.

[7] Śliwa-Tytko P, Kaczmarska A, Lejman M, Zawitkowska J (2022). Neurotoxicity Associated with Treatment of Acute Lymphoblastic Leukemia Chemotherapy and Immunotherapy. *International Journal of Molecular Sciences*.

[8] Teng C, Frei CR (2022). Delirium Associations with Antibiotics: A Pharmacovigilance Study of the FDA Adverse Event Reporting System (FAERS). *Drugs - Real World Outcomes*.

[9] Yang XL, Chen YJ, Ou W, Xie XH (2024). Meropenem-Associated Delirium. *American Journal of Therapeutics*.

[10] Munoz-Gomez S, Gran A, Cunha BA (2015). Meropenem delirium: a previously unrecognized neurologic side effect. *Journal of Chemotherapy (Florence, Italy)*.

[11] Tabulov C, Montoya M, Panzarino V (2024). Meropenem-Induced Delirium in a Hemodialysis-Dependent Adolescent Girl. *Clinical Pediatrics*.

[12] Norrby SR (1996). Neurotoxicity of carbapenem antibacterials. *Drug Safety*.

[13] Naranjo CA, Busto U, Sellers EM, Sandor P, Ruiz I, Roberts EA (1981). A method for estimating the probability of adverse drug reactions. *Clinical Pharmacology and Therapeutics*.

[14] Spriet I, Goyens J, Meersseman W, Wilmer A, Willems L, Van Paesschen W (2007). Interaction between valproate and meropenem: a retrospective study. *The Annals of Pharmacotherapy*.

[15] Baldino F, Geller HM (1981). Sodium valproate enhancement of gamma-aminobutyric acid (GABA) inhibition: electrophysiological evidence for anticonvulsant activity. *The Journal of Pharmacology and Experimental Therapeutics*.

[16] Wang LL, Ren XX, He Y, Cui GF, Liu JJ, Jia J (2022). Pharmacokinetics of Diazepam and Its Metabolites in Urine of Chinese Participants. *Drugs in R&D*.

[17] Kennedy GM, Lhatoo SD (2008). CNS adverse events associated with antiepileptic drugs. *CNS Drugs*.

