# Supplementary material for: Delirium Mistaken for Bipolar Disorder in a Paediatric Oncology Patient: A Case Report
Source: Actas Esp Psiquiatr. 2025 Dec 17;53(6):1448–53. doi: 10.62641/aep.v53i6.2006 (PMC12728545; doi:10.62641/aep.v53i6.2006)
Supplement: Supplementary file 1 [file ActEsp-53-6-1448-1453-s1.zip › Supplementary material 1.docx]

supplementary material 1

1. MRI

Siemens 3.0 Tesla scanner (Allegra, Siemens Medical System, Erlangen, Germany)

1. Vincristine (Vincristine Sulfate for Injection)

lot number: H20043326

Manufacturer: Zhejiang Hisun Pharmaceutical Co., Ltd. Zhuji, Zhejiang, China.

1. Daunorubicin (Daunorubicin Hydrochloride for Injection)

Lot number：H44024361

Manufacturer: Shenzhen Main Luck Pharmaceuticals Inc. Shenzhen, Guangdong, China.

1. l-asparaginase (Asparaginase （Erwinia）for Injection)

lot number：H19993914

Manufacturer: Guangzhou Baiyunshan Mingxing Pharmaceutical Co., Ltd. Guangzhou, Guangdong, China.

1. **Dasatinib （Dasatinib Tablets）**

**Lot number：H20133271**

Manufacturer: Chia Tai Tianqing Pharmaceutical Group Co., Ltd. Lianyungang, Jiangsu, China.

1. electroencephalography (EEG)

Wuhan Greentek Pty. Ltd. Wuhan, Hubei, China.

1. Meropenem (Meropenem for Injection)

lot number：H20030331

Manufacturer: Huanhui Pharmaceutical Co., Ltd. Shanghai, China.

1. Vancomycin (Vancomycin Hydrochloride for Injection)

lot number：H20084268

Manufacturer: Zhejiang Hisun Pharmaceutical Co., Ltd. Zhuji, Zhejiang, China.

1. Diazepam (Diazepam Injection)

lot number：H23021885

Manufacturer: Hayao Group Sanjing Pharmaceutical Co., Ltd. Harbin, Heilongjiang, China.

1. Midazolam (Midazolam Injection)

lot number：H20143222

Manufacturer: Jiangsu Enhua Pharmaceutical Co., Ltd. Xuzhou, Jiangsu, China.

1. Aripiprazole (Aripiprazole Tablets)

lot number：H200061304

Manufacturer: Zhejiang Otsuka Pharmaceutical Co., Ltd. Shaoxing, Zhejiang, China.

1. Valproate (Sodium Valproate Sustained-release Tablets（I）)

lot number：H20010595

Manufacturer: Sanofi (Hangzhou) Pharmaceutical Co., Ltd. Hangzhou, Zhejiang, China.

1. dexamethasone (Dexamethasone Sodium Phosphate Injection)

lot number：H44022090

Manufacturer: Guangzhou Baiyunshan Mingxing Pharmaceutical Co., Ltd. Guangzhou, Guangdong, China.
